# Spironolactone and XPB: An Old Drug with a New Molecular Target

**DOI:** 10.3390/biom10050756

**Published:** 2020-05-13

**Authors:** Ryan D. Gabbard, Robert R. Hoopes, Michael G. Kemp

**Affiliations:** Department of Pharmacology and Toxicology, Wright State University Boonshoft School of Medicine, Dayton, OH 45435, USA; gabbard.21@wright.edu (R.D.G.); hoopes.4@wright.edu (R.R.H.)

**Keywords:** DNA repair, UV radiation, genomic instability, transcription, tumor immunobiology, drug screening, mutagenesis, DNA damage response, viruses, proteolysis

## Abstract

Spironolactone (SP) is commonly used for the treatment of heart failure, hypertension, and complications of cirrhosis by antagonizing the mineralocorticoid receptor. However, SP also antagonizes the androgen receptor, and thus SP has also been shown to be effective in the treatment of acne, hair loss, and hirsutism in women. Interestingly, recent drug repurposing screens have identified new and diverse functions for SP as a simulator of tumor immunosurveillance and as an inhibitor of DNA repair and viral infection. These novel pharmacological effects of SP have all been linked to the ability of SP to induce the rapid proteolytic degradation of the xeroderma pigmentosum group B (XPB) protein. XPB is a critical enzymatic component of the multi-subunit complex known as transcription factor II-H (TFIIH), which plays essential roles in both DNA repair and the initiation of transcription. Given the critical functions for XPB and TFIIH in these processes, the loss of XPB by SP could lead to mutagenesis. However, the ability of SP to promote cancer stem cell death and facilitate immune recognition may counteract the negative consequences of SP to mitigate carcinogenic risk. Thus, SP appears to have new and interesting pharmacological effects that may extend its potential uses.

## 1. Introduction

**Figure 1:**
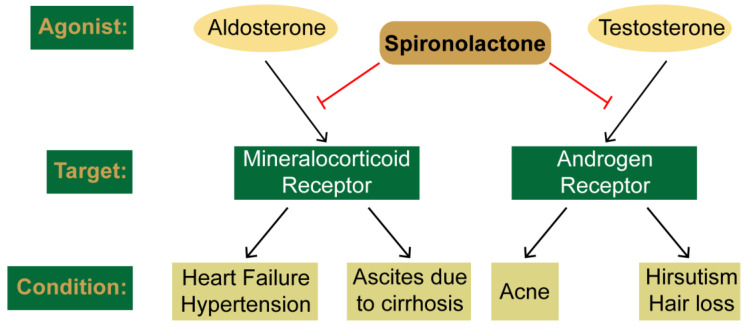
Traditional targets of spironolactone. Spironolactone antagonizes the actions of the aldosterone and testosterone towards their target receptors, the mineralocorticoid and androgen receptors, respectively. Inhibition of these receptors in used to treat the indicate conditions.

The ideal small molecule compound exhibits specificity to a single enzyme or molecular entity to allow for maximum drug efficacy with minimal side effects. However, most pharmacological agents affect multiple biological targets, which can frequently limit the maximum tolerable dose or prevent the continued use of a drug. In some cases, the lack of specificity increases the number of disease states for which a given compound can be clinically employed. Spironolactone (SP) provides a classic example. First used in the treatment of hypertension and heart failure due to its ability to antagonize aldosterone action on the mineralocorticoid/aldosterone receptor, SP was later shown to also target the androgen receptor (Figure 1). This then led to the use of SP in a variety of androgen-induced dermatologic conditions, including acne vulgaris and the excessive hair growth condition hirsutism. Thus, a single agent can be prescribed to treat diverse disorders and disease states.

To capitalize on this lack of drug specificity and to lower the cost of drug therapies, drug repurposing screens are commonly carried out to identify new uses for old drugs [1]. SP again provides a pertinent example. As described in detail below, recent cell-based screens have identified diverse roles for SP in tumor immunotherapy [2], as adjuvants in DNA damage-based cancer chemotherapy [3,4], and preventing viral infection [5]. Interestingly, as will be described below, these novel functions all appear to be associated with the ability of SP to induce the rapid proteolytic degradation of the xeroderma pigmentosum group B (XPB) protein. XPB plays important roles in transcription and DNA repair, and thus the loss of these functions may cause SP to increase mutagenesis and cell death.

In this review, we therefore highlight the current knowledge of SP’s mechanism of action and then discuss recent work that has uncovered new potential uses for SP in immunology, virology, and cancer biology.

## 2. Clinical Uses and Canonical Targets of Spironolactone

### 2.1. SP as a Mineralocorticoid Receptor Antagonist

**Figure 2:**
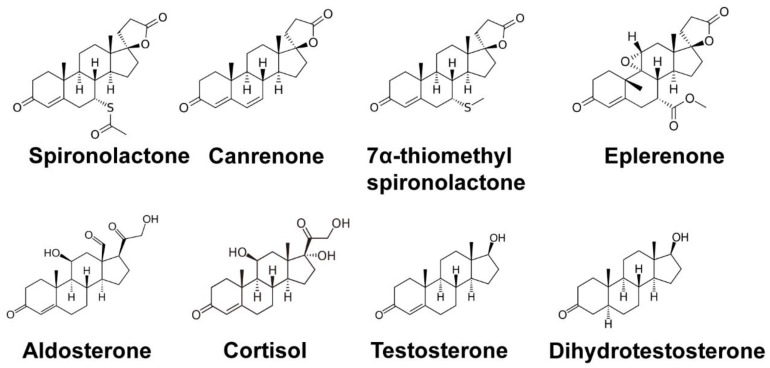
Structures of spironolactone and related molecules. Chemical structures are provided for spironolactone, its metabolites canrenone and 7α-thiomethylspironolactone, the mineralocorticoid receptor antagonist eplerenone, the mineralocorticoid agonist aldosterone, the related molecule cortisol, and the androgen receptor agonists testosterone and dihydrotestosterone.

The mineralocorticoid aldosterone is secreted by the adrenal glands and is thought to contribute to a number of pathological conditions, including myocardial fibrosis, endothelial dysfunction, and vascular inflammation. These processes are involved in the development of heart failure, which is a leading cause of morbidity and mortality throughout the world. In the late 1950s and early 1960s, animal and human studies demonstrated that the synthetic mineralocorticoid SP could antagonize the renal excretory effects of aldosterone [6,7] and reduce arterial pressure in patients with hypertension [8,9]. Due to mineralocorticoid receptor (MR) antagonism in the kidney, SP results in increased excretion of sodium and water to lower fluid retention and lessen pressure on the heart. Both SP and the related mineralocorticoid antagonist (MRA) eplerenone (Figure 2) have been shown to reduce total and cardiovascular mortality in heart failure patients when administered along with other inhibitors of the renin–angiotensin–aldosterone (RAAS) system [10,11]. Canrenone, a metabolite of SP (Figure 2), also acts as an MRA and thus can be used in some cases. SP is also the primary drug of choice for initially treating ascites due to cirrhosis and can be used in doses up to 400 mg per day to achieve optimal sodium excretion [12]. Lastly, both SP and eplerenone have been used in the treatment of diabetic kidney disease [13], where these MRAs can protect against organ damage. Thus, there are multiple related conditions in which SP may have therapeutic benefit.

### 2.2. SP as an Androgen Receptor Antagonist

Though SP has been successfully used for hypertension and heart failure, the feminizing effects of the drug in males, such as gynecomastia, were also noted [14]. During the 1970s, SP was found to antagonize androgen receptors (ARs) [15,16], which explains the unique effects of SP in males. However, AR antagonism ultimately led to the use of SP in a variety of for androgen-associated skin conditions (Figure 2), including acne and hair growth dysfunction [17,18]. Acne is best understood as a disease of excessive sebum production which consequently leads to blocked pilosebaceous follicles by way of undifferentiated keratinocytes, resulting in a lipid-rich environment for Propionibacterium acnes to thrive [19]. An adaptive and innate immune reaction is triggered, resulting in the recruitment of leukocytes to the blocked pilosebaceous follicles and causing the erythematous reaction seen in acne. Dihydrotestosterone (DHT) binds to the androgenic receptors within the pilosebaceous follicles prompting the release of sebum, a lipid-rich material that coats the hair follicles. Increased sebum levels of androgens seen in adolescent males and females undergoing puberty results in acne vulgaris commonly seen within this population. Thus, the result of blocking these androgens from binding to ARs within the sebocytes by SP would lead to less sebum production and a reduction in inflamed pilosebaceous glands. 

A plethora of studies have demonstrated the safety and tolerability of spironolactone at low doses for acne treatment in women [17,18,20]. These doses typically range from 25 to 200 mg per day in one to two divided doses. However, spironolactone has seen a dramatic increase in dosage among two particular demographics: patients with female pattern hair loss (FPHL) and transgender women. SP has been shown to arrest hair loss progression and stimulate partial hair regrowth. The typical dose for non-transgender women being treated for either acne or hair loss is typically 200 mg; however, doses as high as 400 mg/day have been reported without any profound effects [21]. The two most commonly used anti-androgen therapies for feminizing hormone therapy are cyproterone acetate (CPA) and spironolactone. However, despite both hormone therapies having a similar mechanism of action, which involves inhibiting the binding of androgens to androgen receptors and reducing androgen biosynthesis, studies have shown a possible link between CPA and liver toxicity [22]. For this reason, CPA is not currently approved in the United States, making spironolactone the more popular of the two worldwide. Current practice guidelines have established 400 mg/day as the safety cut-off for male-to-women spironolactone regimens [23].

In addition to antagonizing the androgen receptor, SP has also been reported to interfere with a cofactor of cytochrome P450 necessary for testosterone synthesis, increase the level of sex hormone binding globulin, and reduce 5α-reductase activity [24,25]. Thus, there may be other mechanisms by which SP may impact androgen signaling in vivo.

## 3. Identification of Novel Pharmacological Effects of Spironolactone

**Figure 3:**
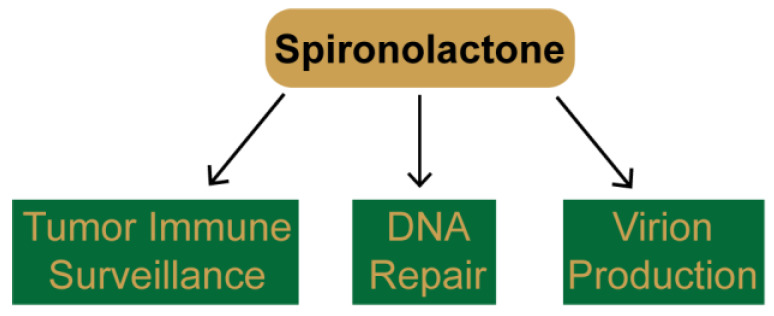
New pharmacological effects of spironolactone uncovered during high-throughput drug screening. Using a variety of different assays, several groups have discovered new functions for spironolactone, including in improving immune recognition by natural killer cells, inhibition of nucleotide excision repair and double-strand break repair, and inhibition of viral transcription and production.

High-throughput screens of small molecule compounds, including FDA-approved drugs, have emerged as a promising way to identify novel modulators of protein function to re-purpose existing drugs for the use in new disease states, such as cancer [1]. As will be described below, several different screens have independently identified SP as a potential drug candidate for a wide range of uses, including tumor immunotherapy, DNA damage-based cancer chemotherapies, and as an anti-viral agent (Figure 3). 

### 3.1. SP Promotes Tumor Cell Recognition by Natural Killer Cells

One mechanism by which the immune system detects abnormal cells in the body to avoid tumorigenesis is via natural killer (NK) and cytotoxic T cells, which recognize NKG2D ligands (NKG2DLs) expressed on the surface of tumor cells and then targets the cells for destruction. Interestingly, SP was uncovered in a screen of 5600 bioactive compounds that up-regulate the expression of the NKG2DL ULBP2 in 293T cells [2]. SP was subsequently shown to increase NKG2DL expression in several different colorectal carcinoma cell lines in a dose-dependent manner and to enhance cell lysis by NK cells in vitro. Similar results were obtained using a xenograft model in vivo in which SP was co-injected along with tumor and NK cells into immunodeficient mice. Moreover, pre-treatment of mice with SP prior to cancer cell implantation was also able to inhibit tumor development. Furthermore, SP treatment was shown to prevent polyp development in a mutant mouse strain (ApcMin/J mice) that is highly susceptible to intestinal neoplasia [2]. Evidence showing that the MR receptor antagonists eplerenone and the SP metabolite canrenone (Figure 2) did not enhance NKG2DL expression indicated that MR antagonism is likely not the mechanism by which SP enhances NK cytolysis. Lastly, the authors identified the DNA damage response kinases ATM (ataxia telangiectasia-mutated) and CHK2 (checkpoint kinase 2) and the nuclear hormone receptor RXRγ (retinoid X receptor γ) as being critical to NKG2DL upregulation and the antitumor function of SP. Thus, these results suggest a possible role for SP in cancer prevention and treatment.

### 3.2. SP Inhibits DNA Repair and Sensitizes Cancer Cells to DNA Damaging Agents

A second high-throughput screen using a library of more than 1200 FDA-approved small molecule drugs identified SP as an inhibitor of UV photoproduct removal in cultured cells [3]. UV photoproducts are solely removed by the nucleotide excision repair (NER) system in human cells [26], and this versatile DNA repair system is capable of targeting many types of DNA adducts, including those caused by cancer-causing polyaromatic hydrocarbons and anti-cancer DNA alkylating agents such as cisplatin. Importantly, the investigators found that pre-treatment of cancer cells with SP, but not the related MR antagonist eplerenone (Figure 2), completely inhibited UV photoproduct removal from DNA and sensitized cancer cells to the lethal effects of both UV radiation and cisplatin. SP alone did not significantly impact cell viability, and thus, this work suggested that SP could be a novel NER inhibitor to improve the effectiveness of platinum-based chemotherapy regimens.

Using the same chemical library and an assay to monitor the specific repair of DNA double-strand breaks (DSBs), Shahar et al. similarly uncovered SP as an inhibitor of the homology-directed repair (HDR) mode of DSB repair [4]. SP was shown to inhibit HDR to an extent similar as an ATM kinase inhibitor and to impair the recruitment of HDR proteins to DSBs. HDR-defective cells are frequently hypersensitive to a variety of chemotherapy drugs, and thus SP was shown to enhance cell killing by both the DNA damaging drug phleomycin, mitomycin C, and a PARP (poly-ADP-ribose polymerase) inhibitor. Lastly, SP was shown to inhibit the growth of HeLa cells implanted into immunodeficient mice. An additional, more recent study reported that cancer stem cells treated with SP may be particularly sensitive to DNA damage in vitro and in vivo and be predisposed to undergo cell death [27]. Thus, SP may be useful in the treatment of cancers with a variety of traditional DNA damaging drugs that induce DSB formation.

### 3.3. SP Inhibits Viral Gene Transcription

A final, functionally distinct screening assay that discovered SP instead dealt with transcription of virus-encoded genes and virion production. Verma et al. were focused on identifying small molecules that inhibit the function of the Epstein–Barr virus (EBV) SM protein [5], which is critical for lytic EBV replication. The investigators found that while SP robustly inhibited EBV production by epithelial cells, its metabolite canrenone had only a modest effect, and the metabolite TMS (7α-thiomethyl-SP) and related MR antagonize eplerenone (Figure 2) were completely inactive. Analyses of the different biochemical steps of EBV production revealed that SP does not impact viral DNA replication but instead specifically impacts the expression of an essential SM-dependent late lytic capsid gene. Thus, SP emerged from this work as a novel anti-herpesvirus drug candidate that acts at a distinct step from traditional anti-viral drugs that typically inhibit viral DNA synthesis.

**Figure 4:**
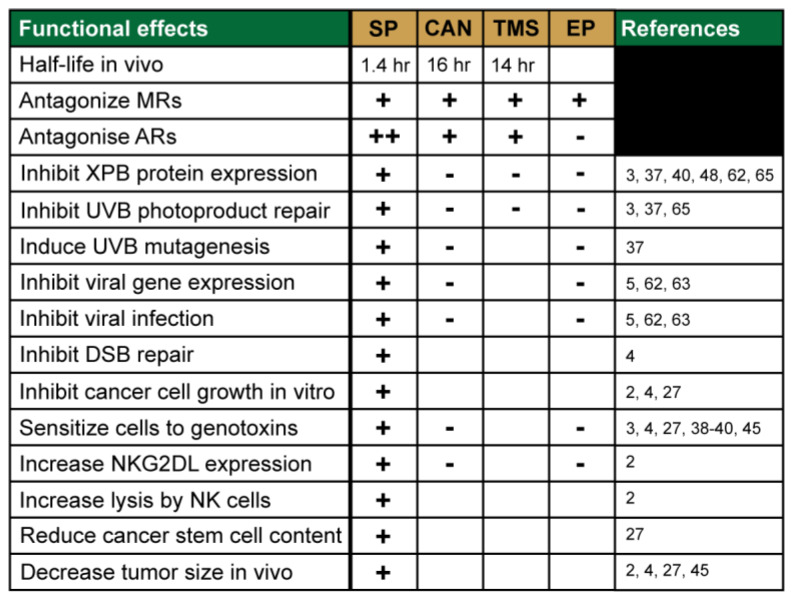
Specific functional effects of spironolactone. The table provides an overview of the different effects that spironolactone, its metabolites, and the related compound eplerenone have been demonstrated to have in different in vitro and in vivo biological systems.

Thus, SP appears to target many different biochemical processes and systems in cells and may impact several disease states and treatment protocols. The variety of functional effects of SP identified in these screens and in subsequent follow-up studies are provided in Figure 4.

## 4. The XPB Protein as a Novel Target of Spironolactone

### 4.1. Identification of the DNA Repair Protein XPB as a Target of SP

The disparate studies highlighted above may suggest that SP has many new targets in cells besides the MR and AR. However, the study by Alekseev et al. provided the first indication that a single protein may explain the diverse effects of the drug. As described above, the authors noted that SP inhibited the removal of UV photoproducts from genomic DNA [3]. This process solely occurs via the NER machinery in human cells and requires nearly 20 polypeptides spread among 6 different core factors [26]. By examining the protein levels of these NER factors, Alekseev et al. observed that only one protein, XPB (xeroderma pigmentosum group B), was rapidly and specifically lost upon SP treatment [3].

**Figure 5:**
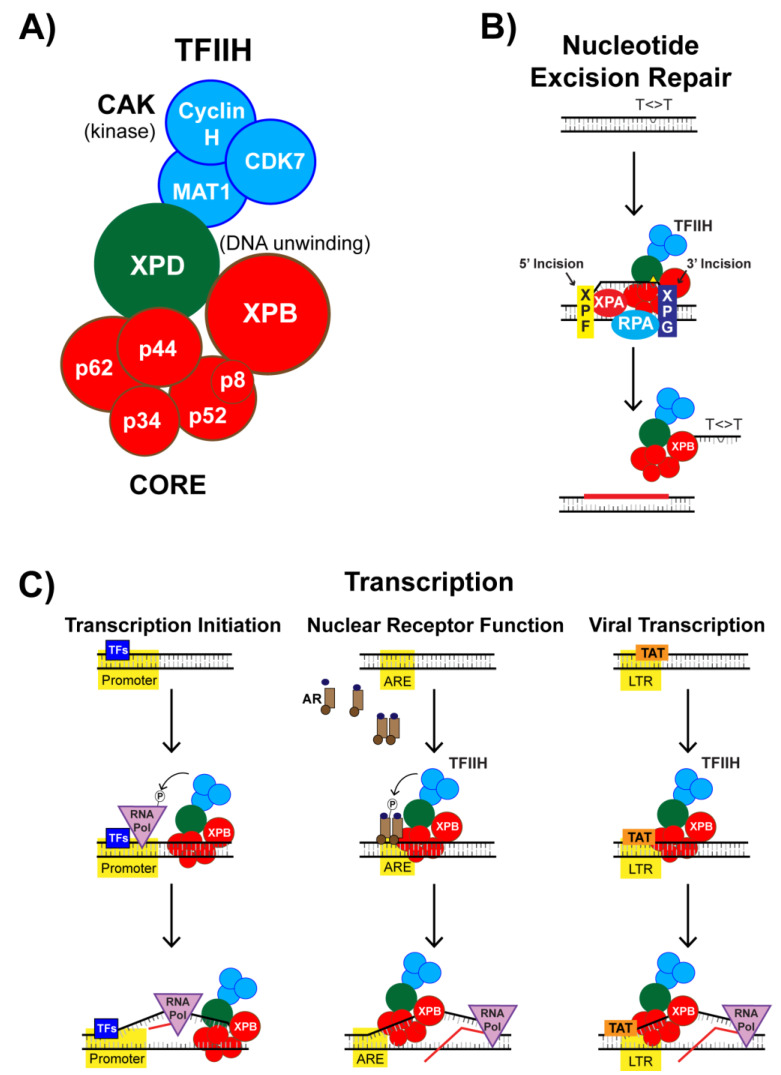
TFIIH structure and function. (**A**) TFIIH (transcription factor II-H) is a 10-subunit protein complex with multiple enzymatic activities, including kinase activity from its cyclin-dependent kinase-activating kinase sub-complex and DNA unwinding activity from the XPD and XPB subunits. (**B**) TFIIH functions in nucleotide excision repair (NER) to unwinding DNA so that the XPF and XPG endonuclease can incise the damage strand of DNA 3′ and 5′ to the lesion, respectively, which releases the damage-containing DNA oligonucleotide in complex with TFIIH. (**C**) TFIIH also functions in general transcription initiation (**left**), nuclear receptor-dependent transcription (**middle**), and in viral transcription (**right**).

XPB is mutated in humans with the disease xeroderma pigmentosum, who are photosensitive and generally exhibit higher rates of skin cancer [28,29]. XPB exists as a component of a larger protein complex known as TFIIH (transcription factor II-H) [30,31,32] (Figure 5A). TFIIH is essential for the removal of UV photoproducts and other bulky adducts from DNA by the NER machinery. In NER, DNA lesions are removed from DNA in the form of small DNA oligonucleotides approximately 30 nt in length via a dual incision mechanism [26] (Figure 5B) that remain bound to TFIIH following their excision [33,34]. For the XPF and XPG endonucleases to incise at sites bracketing the lesion, the DNA must be unwound around the lesion. Though the XPD subunit of TFIIH possesses the primary helicase activity responsible for unwinding DNA around the lesion, the ATPase activity of XPB is thought to help to anchor TFIIH to the chromatin [35] and to separate the two strands of DNA around the lesion so that XPD can bind, further unwind DNA, and verify that a lesion is present [30,36]. Thus, in the absence of XPB function due to genetic mutations or SP treatment, the DNA lesions are unable to be properly processed and excised from the genome. This lack of lesion removal therefore provides an explanation as why NER is inhibited by SP and why SP was found to sensitize cells to UV radiation and cisplatin [3,37,38,39]. Given the wide variety of DNA damaging agents that induce lesions targeted for removal by NER, SP could be used to improve the effectiveness of DNA damaging anti-cancer drugs in many different cancer types. Indeed, a preclinical study showed that sensitivity of treatment of multiple myeloma cells to the alkylating agent melphalan could be improved by co-treatment with SP [40]. However, many chemotherapies induce side effects that could be worsened by treatment with SP. For example, a recent study monitoring cisplatin-induced peripheral neuropathy in mice demonstrated that SP worsened this condition [41].

XPB has not been reported to be involved in DNA double-strand break (DS) repair, and thus the discovery that SP inhibited homology-directed DSB repair [4] may be somewhat more difficult to reconcile. However, mutations in the XPD component of TFIIH have been reported to interfere with transcription-associated recombination in mammalian cells [42] and to cause replication fork breakage and restart in budding yeast [43]. Moreover, mass spectrometric analyses of proteins enriched at replication forks stalled by hydroxyurea treatment revealed the presence of several components of TFIIH, including XPB [44]. Furthermore, a recent study found that SP sensitized cancer cells to the nucleoside analog gemcitabine and the PARP inhibitor osimertinib both in vitro and in mice in vivo [45]. Both compounds do not induce DNA damage directly and can instead interfere with replication fork progression and stability that could collapse to generate DSBs. Thus, it is possible that XPB-containing TFIIH complexes function in some capacity at stalled replication forks, double-strand break repair intermediates, or at collisions between the replication and transcription machineries, all of which may generate DNA structures that require the DNA unwinding functions of TFIIH. However, further studies are clearly needed to better understand how SP inhibits other genome maintenance pathways besides NER.

### 4.2. SP Impacts TFIIH Function in Transcription Initiation

As its name implies, TFIIH also functions to promote the initiation of gene transcription via two key enzymatic functions [30,31]. The first and potentially most important function involves the XPB subunit of TFIIH, which promotes the opening of DNA at gene promoters via ATP-dependent DNA translocase activity [46,47]. This DNA strand separation is necessary for transcription initiation so that a single-stranded template DNA can engage the active site of RNA polymerase II for transcription initiation (Figure 5C, left), and thus, XPB is thought to act as a molecular wrench in this context to open promoter DNA. A second activity of TFIIH is found with the cyclin-dependent kinase (CDK)-activating kinase (CAK) subcomplex (Figure 5A), which is composed of CDK7, Cyclin H, and MAT1. CDK7 promotes transcription via the phosphorylation of several proteins, including RNA Polymerase II, the positive transcription elongation factor b (p-TEFb), and various transcription factors.

Consistent with the general transcription function of XPB and TFIIH, other experimental evidence indicates that SP can inhibit gene expression in vitro and in vivo. For example, though antagonism of the MR receptor by SP is the classical model for how SP improves endothelial dysfunction and survival in heart failure, recent work suggests that SP may prevent inflammation in target tissues by interfering with the expression of pro-inflammatory gene products [48]. For example, Elinoff et al. showed that SP, but not the MR antagonist eplerenone, suppressed NF-κB and AP-1 reporter activity in cells in vitro independent of the MR, AR, or glucocorticoid receptor (AR), and this phenotype could be counter-acted by overexpression of XPB [48]. SP treatment was further shown to be associated with low levels of RNA polymerase II and XPB at the IL-8 promoter in TNFα-treated pulmonary artery endothelial cells in vitro. Lastly, additional studies demonstrated that SP decreased XPB protein levels in whole lungs of a rat model of pulmonary hypertension and reduced the levels of several inflammatory cytokines in the serum of pulmonary arterial hypertension patients. Thus, via inhibition of TFIIH function in transcription initiation, SP may interfere with the transcriptional induction of pro-inflammatory genes in hypertension.

### 4.3. Nuclear Receptor-Dependent Transcription is Regulated by TFIIH and SP

Upon binding to their cognate ligand, nuclear receptors (NRs) facilitate the transcription of specific gene networks [49]. NRs directly associate with transcription factors, including with TFIIH, to mediate gene transactivation (Figure 5C, middle). NRs can also be targeted for phosphorylation by CDK7 within TFIIH to promote the recruitment of other transcriptional co-activators to enhance transcription or to terminate the ligand response [49]. For example, TFIIH promotes both AR-dependent transactivation at promoters and AR turnover [50]. These results suggest that some of the anti-androgen activity of SP may not only be due to direct antagonism of the receptor but also to altered transcription initiation at AR-regulated genes.

In addition to inhibiting transcription, other NR promoter activities may be stimulated by SP. For example, the work described above showing that SP induced an up-regulation of NKG2DL expression in colorectal cancer cells identified the nuclear hormone receptor (RXRγ) as responsible for this upregulation [2]. Reporter assays confirmed that SP treatment increased RXRγ promoter activity. However, the precise mechanism by which affects NKG2DL expression may be complex, as loss of RXRα instead increased NKG2DL expression. Thus, the regulation of NKG2DLs by RXR family members and the impact that SP has on modulating transcriptional responses remained to be better defined. Nonetheless, the genomic stress response kinases ATM and ATR had previously been reported to be required for NKG2DL expression [51], and Leung et al. observed that ATM-CHK2 pathway was specifically activated in an RXRγ-dependent manner in colorectal cancer cells [2]. Thus, though questions remain regarding how SP induces NKG2DL upregulation, the involvement of the NR RXRγ is consistent with the hypothesis that SP may be working through the function of XPB/TFIIH on NR function in gene transactivation.

### 4.4. SP Impacts TFIIH Function in Viral Transcription

It is well-recognized that many viruses co-opt the human host cell replication and transcription machinery to enable the production of more virions. For example, the HIV-1 transcription activator Tat protein is necessary for transcription of viral genes [52,53]. In addition to binding to the transactivation response element (TAR) in the nascent viral RNA [54], Tat is also known to interact with several basal transcription factors at the HIV-1 promoter to stimulate transcription [55], including by binding directly to the CAK complex of TFIIH [56,57]. Tat also interacts with p-TEFb to promote transcription elongation by enhancing p-TEFb recruitment and release of paused RNA polymerase II at the HIV-1 promoter (Figure 5C, right).

Interestingly, XPB has previously been reported to impact HIV-1 infection. Though early studies indicated XPB may protect against retroviral infection [58,59], other screening work suggested that XPB was required for HIV-1 production [60,61]. Based on the finding that SP caused the rapid degradation of XPB [3], Lacombe et al. used SP treatment to determine whether SP could affect HIV-1 transduction in vitro [62]. The authors found that SP, but not eplerenone, caused a loss of XPB protein expression in T cells in vitro and inhibited HIV-1 and HIV-1 transduction. Consistent with the known role of XPB in transcription, SP was also observed to inhibit Tat-dependent promoter activity. A recent study further suggested a similar effect for SP in T-cells infected with human T-cell lymphotropic virus type 1 (HTLV-1) [63], in which the HTLV-1 Tax oncoprotein interacts with XPB to promote TFIIH-dependent transcription at the viral promoter. Along with the previous screening study that identified SP as an inhibitor of EBV SM protein-dependent transcription and virus production [5], these results imply a common mechanism for SP function as an anti-viral compound: SP-induced loss of the XPB protein likely interferes with the ability of viral proteins to co-opt TFIIH and the host cell transcription machinery to transcribe viral genes.

## 5. SP Promotes the Rapid Proteasomal Degradation of XPB

**Figure 6:**
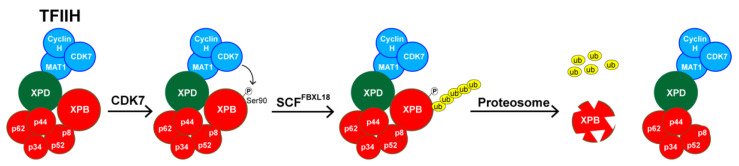
Schematic of the spironolactone-induced XPB degradation pathway. In response to spironolactone treatment, the XPB subunit of TFIIH becomes phosphorylated on Ser90 by CDK7, which then promotes poly-ubiquitination of XPB by the SCF^FBXL18^ E3 ubiquitin ligase to induce degradation by the proteasome.

The mechanism by which SP induces the loss of XPB protein was initially investigated by Alekseev et al. and was shown to occur rapidly after addition of SP to the culture medium and to be independent of XPB mRNA levels [3]. Several other groups have confirmed these general findings in different cell types in vitro and in tissues ex vivo and in vivo [37,48,62,64,65]. The effect of SP on XPB protein levels was also reversible, as XPB protein levels could be fully restored within a few hours of withdrawing the drug in vitro. Alekseev et al. demonstrated that the loss of XPB was mediated by ubiquitination and the proteasome [3]. Recently, SCFFXL18 was identified in an siRNA library screen as the E3 ubiquitin ligase responsible for XPB poly-ubiquitination and degradation [64] (Figure 6). Interestingly, Ueda et al. also identified CDK7 kinase activity as required for this process. Mutation of a putative CDK7 target site in XPB (Ser90) to non-phosphorylatable Ala residue abolished the ability of SP to destabilize XPB. This region of the protein may be important for the degradation of XPB by SP, as a clinically observed Phe99Ser mutation in XPB, which causes severe defects in NER [66], made the XPB protein resistant to degradation by SP [48]. Whether CDK7 within the same TFIIH complex or CDK7 in a free CAK complex is responsible for this process remains to be examined. Nonetheless, these data provide some initial insights into how SP induces XPB degradation.

## 6. Implications for SP in the Skin and Potential Risks of Carcinogenesis

Though the traditional use of SP is as an oral medication, there is research underway investigating ways to allow for topical administration of the drug onto skin [67,68,69,70,71]. This is particularly relevant for skin conditions like acne [72] and hair loss, where the anti-androgen effects of SP largely limits its use to females. However, the topical administration of SP may also be useful as an MR antagonist. For example, glucocorticoids are frequently used to treat inflammatory skin diseases, but their use may be limited by side effects including skin atrophy and an inhibition of wound healing. These side effects appear to be due to glucocorticoids stimulating not only the GR receptor but also the MR receptor. Thus, MR antagonists such as SP have been found in experimental studies to counteract some of these negative effects of glucocorticoids [73,74,75]. Indeed, clinical studies in humans have demonstrated that SP and its related MR antagonists limit glucocorticoid-induced skin atrophy and improve re-epithelialization of wounds [76,77].

The observation that SP promotes the degradation of the critical DNA repair protein XPB might indicate that patients taking the drug orally or topically are at an elevated risk of skin cancer development in regions of sun-exposed skin. Though in vitro studies with keratinocytes and ex vivo studies with skin explants showed that SP can deplete these cells and tissues of XPB, inhibit UV photoproduct removal, and increase mutagenesis [37,65], epidemiological studies have found no evidence that SP use is associated with increased cancer risk of any cancer type, including in the skin [78]. There are several possible explanations for these observations, including the fact that orally administered SP is rapidly metabolized (Figure 3) by the liver into compounds [79] such as canrenone and 7α-thiomethylspironolactone (TMS) (Figure 2) that do not affect XPB protein levels [37,64]. Moreover, as described above, the loss of XPB could cause cancer stem cells to be predisposed to undergo cell death after DNA damage [27] or to increase the recognition of mutant cells by NK cells [2]. Thus, SP may have both pro-mutagenic and anti-cancer properties that counteract one another such that the net effect is no elevated risk of carcinogenesis. However, it should be noted that the metabolism of SP in the skin after topical treatment has not been extensively examined [68]. Thus, topical application of SP onto the skin or the use of SP in male-to-female transgender individuals, which frequently employ much higher doses of SP, could raise some concerns that XPB may be significantly depleted in epidermal cells. Thus, examining XPB levels in areas of skin treated topically with SP may be worthy of investigation. Moreover, populations using high doses of SP should avoid excess UV exposure such as from artificial tanning bed sources.

## 7. Conclusions

In addition to the classical function of SP as an antagonist of mineralocorticoid and androgen receptors, several independent screens identified SP as a novel pharmacological agent for use in preventing tumor development via increased immunosurveillance, inhibiting DNA repair, sensitizing cancer cells to undergo cell death, and in blocking viral transcription and virion production. Thus, in addition to the many diverse clinical disorders ranging from heart failure to acne that currently employ SP as a treatment, there may be other potential uses for SP in new fields of medicine. Because the DNA repair/transcription protein XPB appears to be the major target for SP in context of these new studies, some concern may be warranted regarding the risk of mutagenesis and carcinogenesis. Nonetheless, SP provides a fascinating example of how an old drug can possibly be used for new purposes.
